# Obituary

**Published:** 1903-11-15

**Authors:** 


### OBITUARY.

### IN MEMORIAM.

WHEREAS, By the death of our honored and esteemed colleague and leader, Dr. Jonathan Taft, the faculty of the College of Dental Surgery of the University of Michigan feels that it has been sorely bereaved in a personal as well as an official manner. Personally, because of his courteous and cheerful conduct in our official and social relations. His attitude toward us has ever been of the most kind and considerate nature and will remain with us as a precious memory. Officially, we shall miss his wise and experienced counsel and his deep interest in and devotion to our work. He was ever ready to make needed sacrifice of time and talent for his beloved profession, and especially for the school of dental education, to which he gave so many years of valuable service. He labored to make this a leading school for training men to the highest ideals of professional culture, that through its alumni professional standards might be upheld and public service of the highest grade secured.

His personal efforts have ceased forever, but his spirit remains to complete the work he designed.

RESOLVED, That we mourn the loss of our beloved colleague, and by official action record on our minutes this expression of our esteem and honor for one who has labored so patiently and faithfully to accomplish that which appears to us most honorable and meritorious.

To his bereaved family we extend our cordial sympathy and pray for the consolation of Him Who giveth and also taketh away.

Adopted October 23d, 1903, by the Faculty of the College of Dental Surgery of the University of Michigan.

---

WHEREAS, After a long and useful career of sixty years, as practitioner, author, journalist and teacher, death has ended the life work of Professor Jonathan Taft, who was universally loved and respected by the dental profession for his scholarly attainments and high ethical standing.

WHEREAS, In the death of Dr. Taft, our profession has lost an advanced thinker and an able and enthusiastic exponent of the best in Dental Surgery.

BE IT RESOLVED, That the Fraternal Dental Society of St. Louis extend our sincere sympathy to Mrs. Taft in her bereavement, which is the bereavement of the whole profession, and express our high regard for the worth and character of this pioneer, who so ably exemplified the highest ideal of American Dentistry.

Unanimously adopted October 20th, 1903.

W. L. WHIPPLE, E. E. HAVERSTICK,
*President pro tem.* *Secretary.*

